# A Case Report and Review of Literature on Hirayama Disease

**DOI:** 10.7759/cureus.67627

**Published:** 2024-08-23

**Authors:** Ritesh Reddy Baddam, Bhavya Chadalavada, Nikhil Ambati

**Affiliations:** 1 Internal Medicine, Gandhi Medical College, Hyderabad, IND; 2 Neurological Surgery, Nizam's Institute of Medical Sciences, Hyderabad, IND; 3 General Surgery, Gandhi Medical College, Hyderabad, IND

**Keywords:** motor neuron disease, nerve conduction studies (ncs), anterior horn cell disease, neck collar, juvenile monomelic amyotrophy, flexion mri, hirayama's disease

## Abstract

Hirayama disease (HD) is a rare, benign, self-limiting condition that typically affects individuals in their 20s. Although the disease is self-limiting, it can result in functional impairment in those affected. The most common presentation is an asymmetrical, unilateral, or bilateral upper limb weakness with wasting. With an interesting pathogenesis and lack of definitive treatment, HD is an interesting neurological conundrum. Mild symptoms in patients often lead to underreporting of the disease, as individuals may not seek medical attention or may not recognize their symptoms. Most case reports in the literature are from Asia and the Middle East. We report a case of HD in a male patient in his 20s with gradual bilateral upper limb weakness and wasting, confirmed by imaging and nerve conduction studies.

## Introduction

Hirayama disease (HD) is an uncommon neurological condition that affects young men in their early 20s and is characterized by progressive wasting and weakness of C7-T1 innervated muscles of the distal upper limbs. Disease progression spontaneously halts within five years [1]. Rarely, upper motor neuron (UMN) signs such as exaggerated reflexes, increased tone, and sensory and autonomic abnormalities can be seen [2]. We report a case of a 22-year-old male patient with gradual and asymmetric bilateral upper limb weakness and wasting. Imaging and nerve conduction investigations established the diagnosis of HD.

## Case presentation

A 22-year-old Indian male, driver by occupation with no relevant medical history, presented with a 12-month duration of neck pain, weakness, and wasting of the hand and forearm on the right side, which he noticed when he found gripping the steering wheel difficult while driving. Initially, he noticed the weakness and atrophy in the small muscles of his right hand and gradually progressed to involve his right forearm muscles. Over the next two months, he also noticed mild weakness and wasting of muscles in his left hand. He did not have any sensory, autonomic, bladder, or bowel complaints, and lower limb muscles were spared. Past history was negative for trauma, recent fever prior to the onset of symptoms, exposure to toxins or heavy metals, and poliovirus infection, and family history for similar complaints was insignificant.

**Figure 1:**
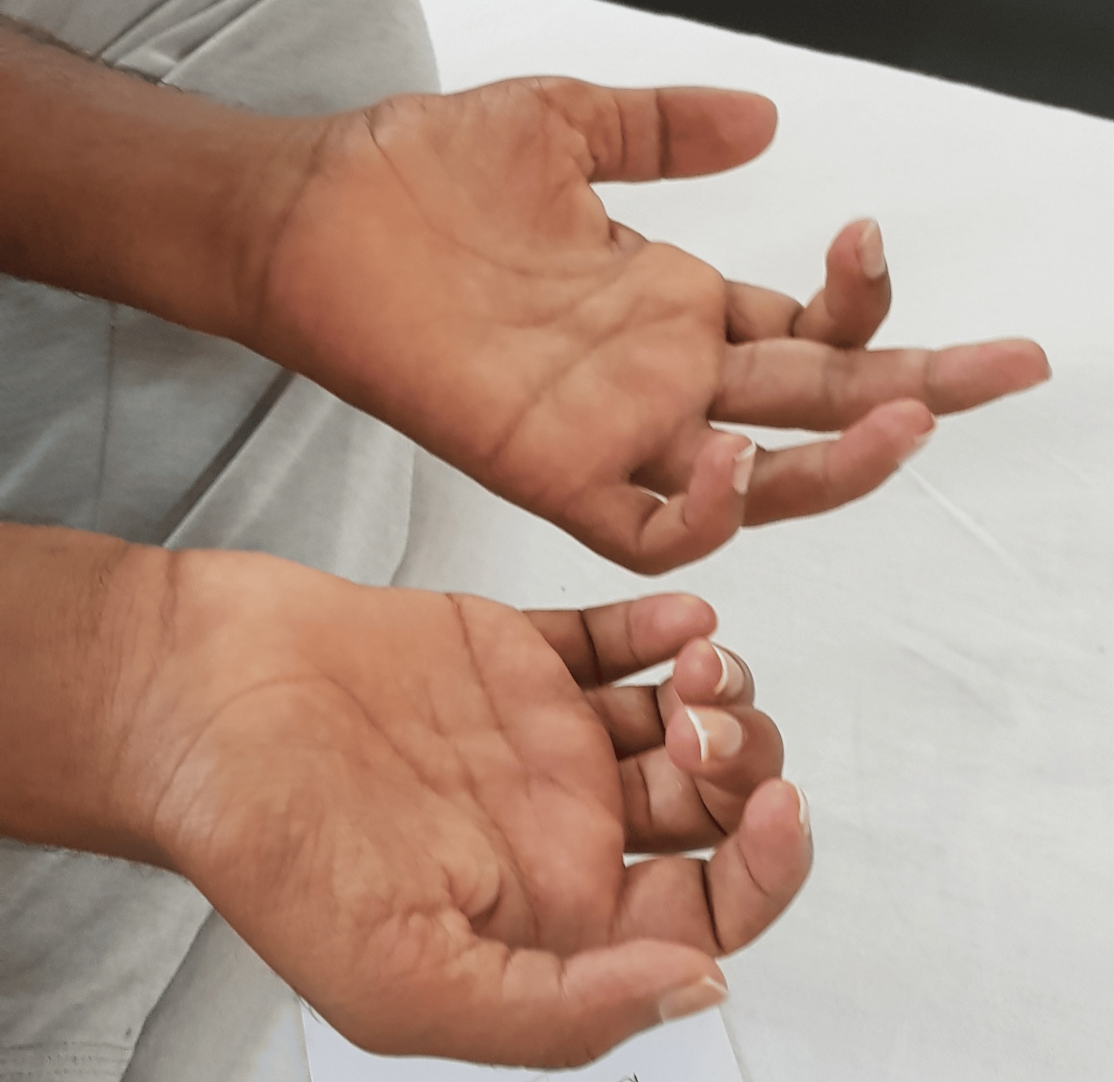
Patient's right and left hands showing intrinsic muscle wasting

**Figure 2:**
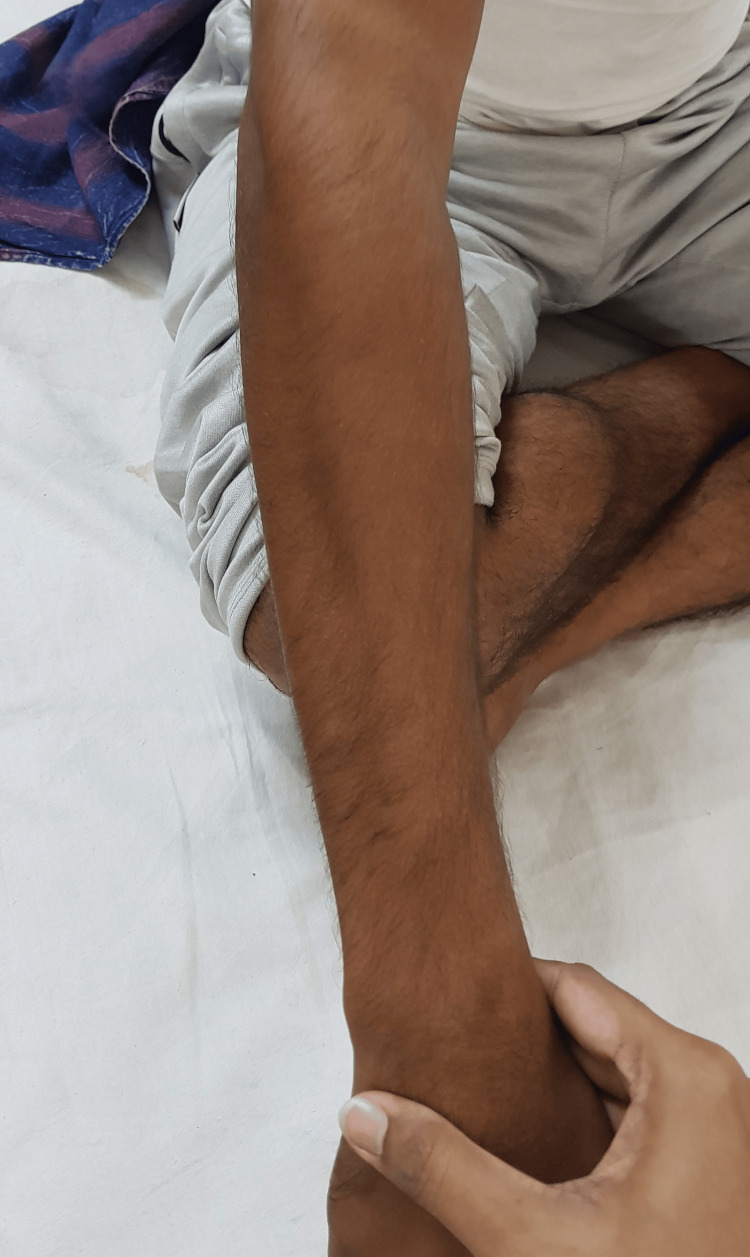
Sparing of the brachioradialis muscle, also known as oblique amyotrophy sign

On neurological examination, both the right and left small muscles of the hand and forearm muscles were weak and atrophied, as shown in Figure 1. The brachioradialis muscle was spared on both sides, as shown in Figure 2, commonly known as the "oblique amyotrophy" sign [2]. All intrinsic muscles of both hands were weak, and palmar grasp was impaired. Wrist flexion and extension were weak. Weakness and wasting of muscles were asymmetrical with the right distal upper limb more affected than the left distal upper limb. Both lower limb and proximal upper limb muscles were normal on examination. Deep tendon reflexes were 2+ all over. There was no involvement of the cranial nerves, sensory system, or cerebellum.

**Figure 3:**
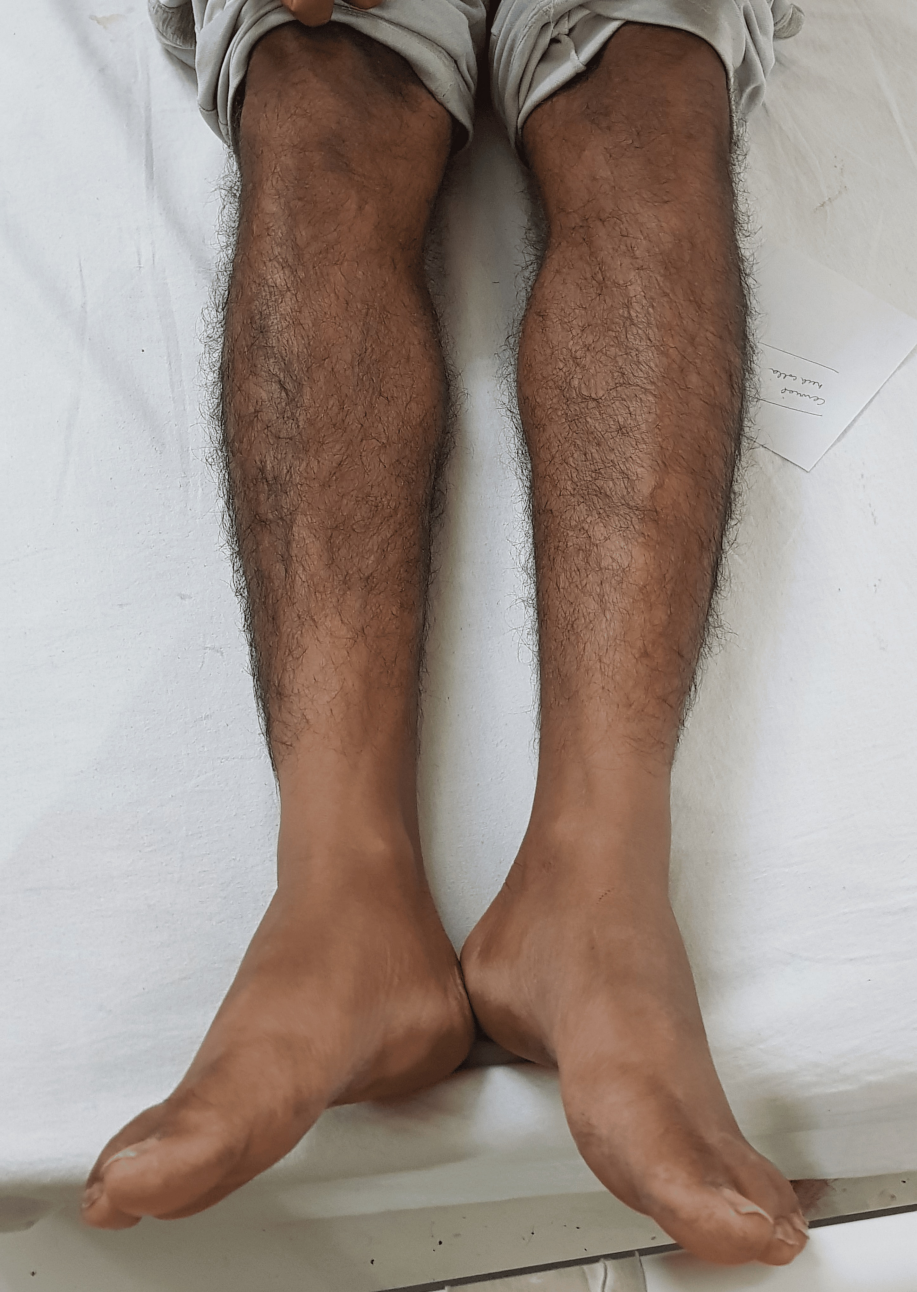
No involvement of the lower limbs

Images of the patient's upper and lower limbs are shown in Figures 1-3.

Investigations

The X-ray of the cervical spine showed a loss of cervical lordosis. Nerve conduction studies (NCS) showed reduced compound muscle action potential (CMAP) amplitude in the bilateral median and ulnar nerves. Conduction velocities, distal latencies, and F-wave latencies were normal. Sensory NCS was normal. Electromyography was suggestive of neurogenic process in right C7, C8, and T1 myotomes with evidence of re-innervation, implying nerve injury or dysfunction, as opposed to muscle damage.

**Figure 4:**
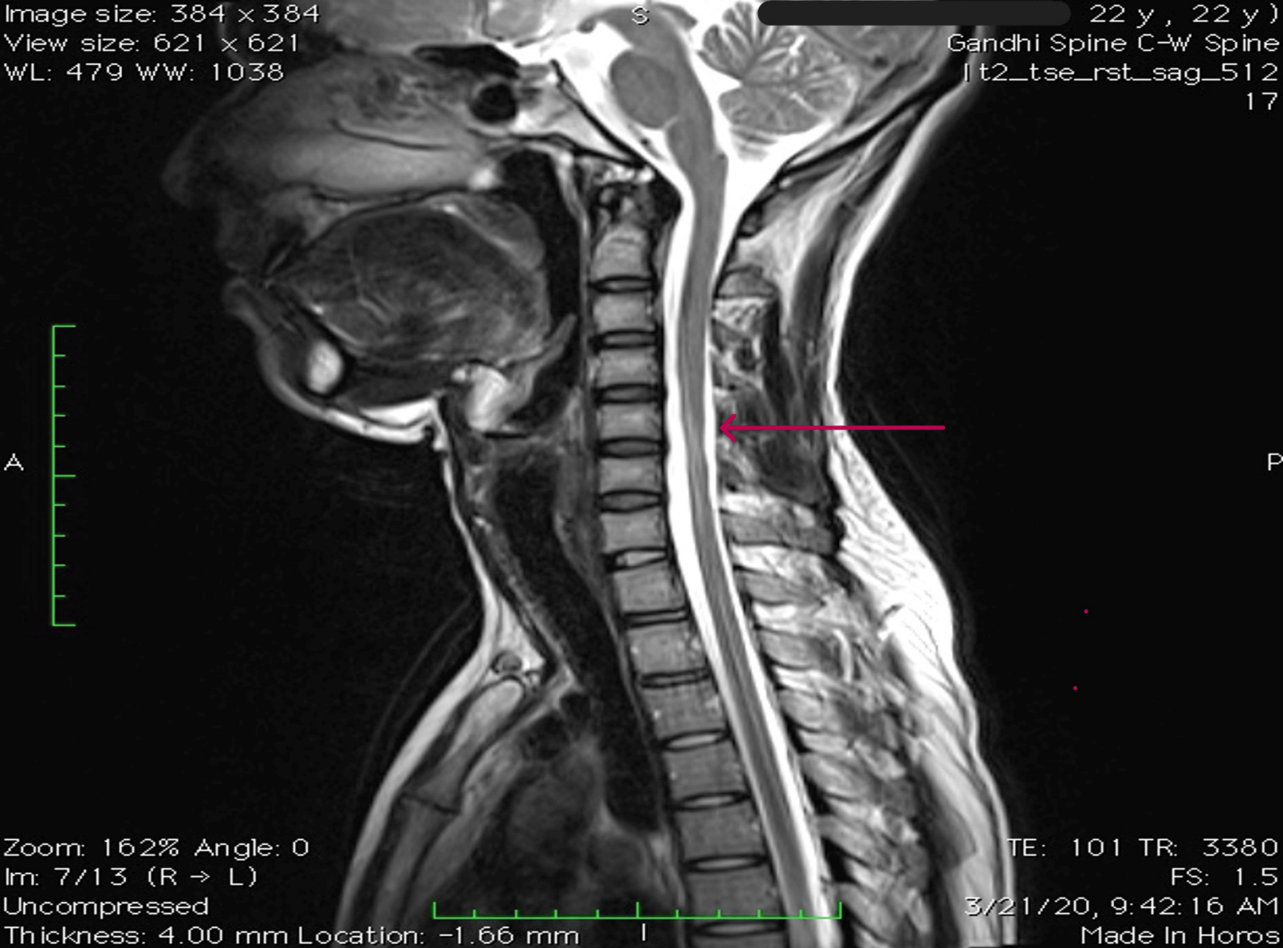
Abnormal T2W sagittal MRI of the neck in a neutral position showing hyperintensity at the site of the maximum forward shift

T2-weighted MRI of the cervical spine with contrast in neutral position showed hyperintensity in anterior horn cells from C6 to T1 bilaterally and localized lower cervical cord atrophy, as shown in Figure 4.

**Figure 5:**
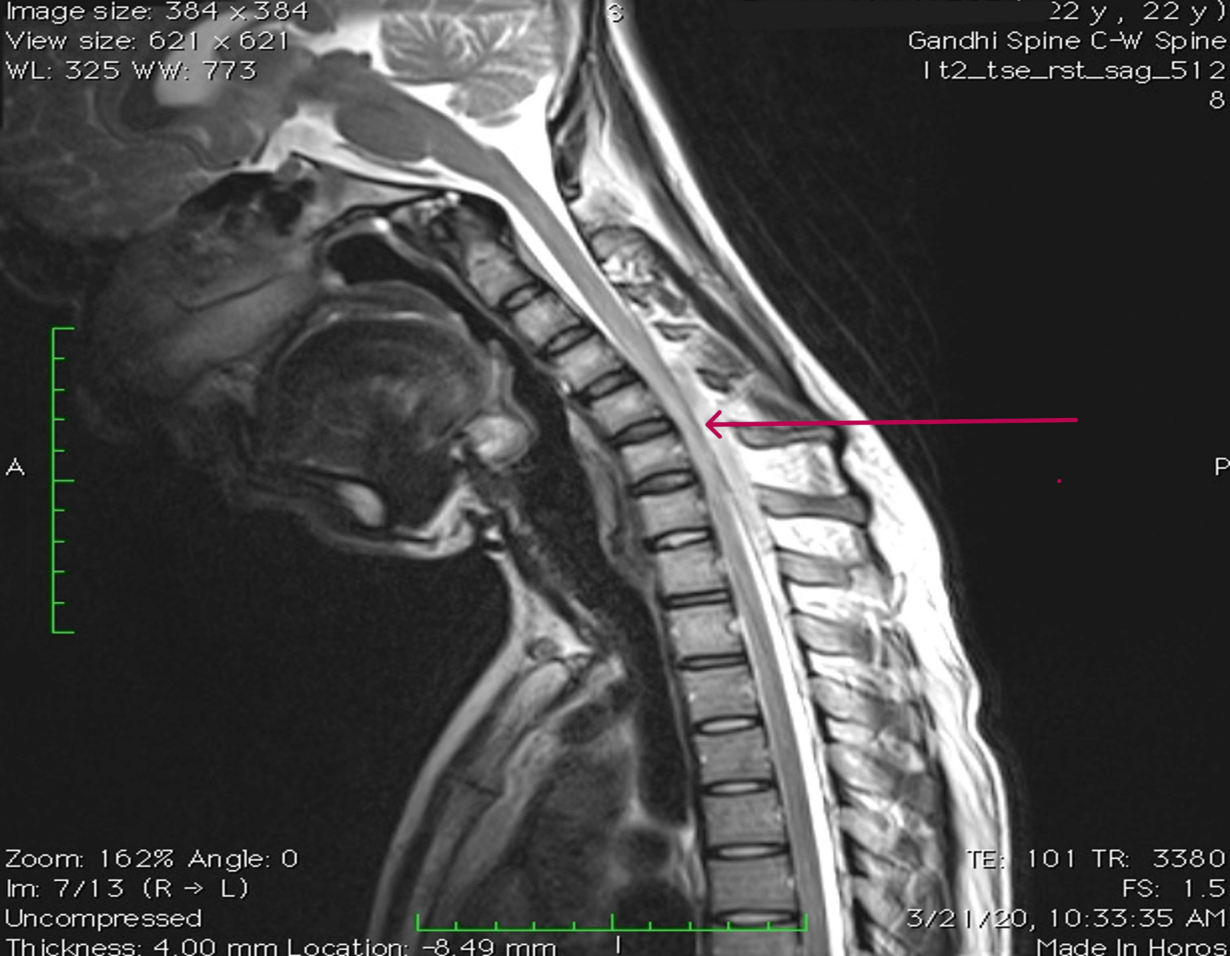
Abnormal T2W sagittal flexion MRI of the neck showing the loss of dural attachment and anterior displacement of the dorsal horn

**Figure 6:**
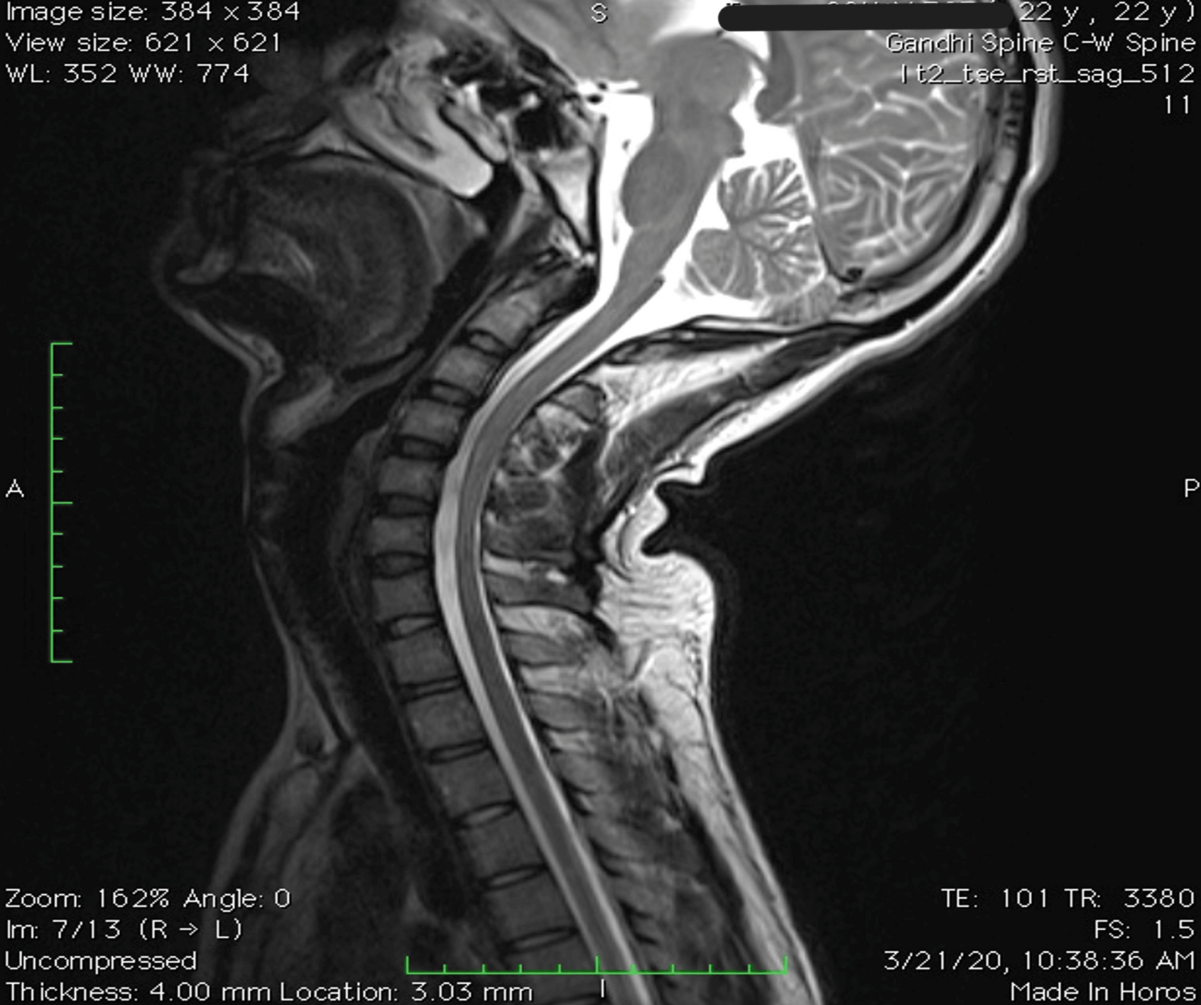
T2W sagittal MRI of the neck in extension; findings seen on flexion MRI are not visible here

**Figure 7:**
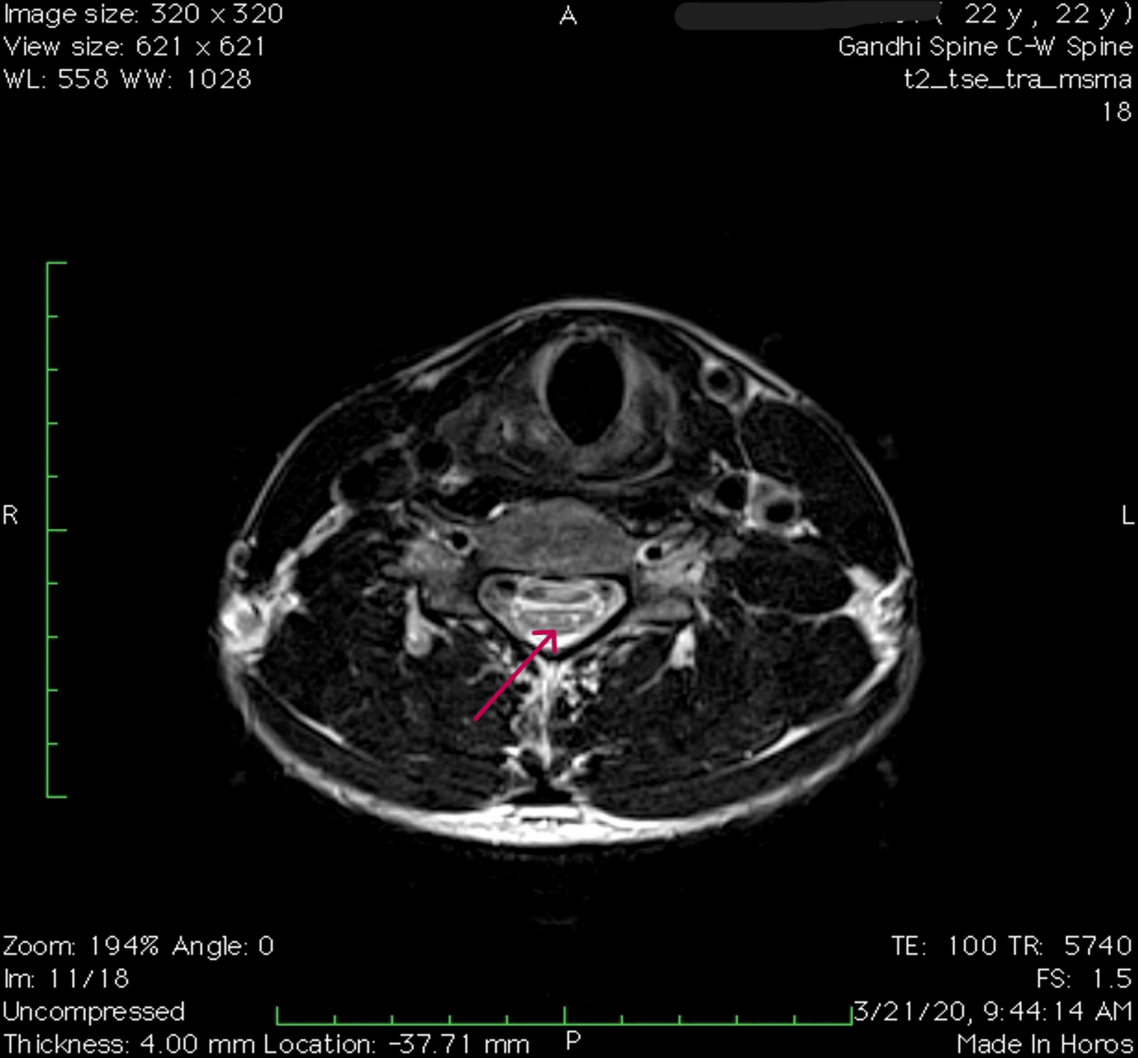
T2W transverse section of the cord showing owl's eye sign (due to involvement of anterior horn cells)

MRI in flexion position showed loss of dural attachment and anterior displacement of the dorsal dura. A T1/T2 crescentic hyperintensity with few epidural flow voids was noted in the posterior epidural space, which showed uniform enhancement post-contrast from C3 to T2 level, causing narrowing of the cord. Posterior dural buckling was noted causing impingement of the anterior cord against the vertebra. MRI images in flexion and extension are shown in Figures 5-6. Bilateral hyperintense foci in anterior horn cells (AHC) of the spinal cord on axial T2-weighted MRI, also known as owl eye sign, are seen in Figure 7.

Complete blood count, erythrocyte sedimentation rate (ESR), renal, liver, and thyroid function tests and creatine kinase were within normal range. Lead levels were normal. Viral serologies for human immunodeficiency virus (HIV), hepatitis B, and hepatitis C were negative.

Overall, the diagnosis of HD was supported by the clinical, NCS, and MRI findings. A cervical collar was recommended to reduce the risk of subsequent spinal cord injury by preventing neck flexion.

## Discussion

In 1959 in Japan, Keizo Hirayama first described HD as a rare disorder of the lower motor neurons [3]. HD is known by several names, including juvenile asymmetric segmental muscular atrophy, juvenile muscular atrophy, monomeric amyotrophy, and benign juvenile brachial spinal muscular atrophy, but the name HD was coined in 1991 and is the most commonly used term. In the Asian population, particularly those from Japan, China, India, Sri Lanka, and Taiwan, the majority of individuals with HD are found. The explanation for the high incidence of cases in Asian nations is still unclear; additional research is necessary to determine whether geographic and ethnic factors play a role. There is a significant male predominance in the male-to-female ratio, which varies greatly between countries [4]. There are case reports of bilateral asymmetrical and bilateral symmetrical HD in literature even though in most cases, unilateral involvement is most seen in patients. In 30% of patients, an asymmetrical bilateral involvement may be seen. In a study of the clinical profile of HD cases in India, bilateral involvement was seen in six out of 11 cases reported by Hassan et al. [5], six out of eight patients reported by Sonwalkar et al. [6], and 30 out of 224 cases reported by Nalini et al. [7].

Pathogenesis

The exact pathogenesis of HD remains a mystery. It is widely believed to occur when flexion of the neck results in the posterior dura moving forward and compressing the spinal cord. The primary pathology, according to autopsy results, is an asymmetry in the cervical anterior horn and anterior roots. In 1987, Hirayama et al. [8] published the first pathological study on a patient 23 years after the disease first appeared. Only the anterior horns of the spinal cord from C5 to T1 showed mild gliosis and necrosis of varying degrees in both large and small nerve cells, with C7 and C8 exhibiting the most degeneration. They proposed that the primary reason is lower cervical cord circulatory insufficiency. The main pathogenetic mechanism of HD is thought to be the marked and frequently asymmetric flattening of the lower cervical cord caused by the posterior wall of the lower cervical dural canal moving forward when the neck is flexed, as demonstrated by the neuroradiology. Hirayama et al. [8] further suggested that dural tightness could be caused by an imbalance in the growth of the spinal cord and spinal canal during the pubertal growth spurt. Anterior dural displacement brought on by cervical flexion causes compression in the anterior spinal artery territory resulting in anterior horn cell infarction in the C7-T1 regions. Toma et al. [9] suggested that the juvenile growth spurt accentuates disproportionate shortening of the dorsal nerve roots, coupled with repeated neck flexion, which can result in microtrauma and relative ischemia of anterior horn cells. This in turn causes atrophy of the muscles innervated by long motoneurons. Additionally, according to Yoshiyama et al. [10], those with HD displayed thicker posterior dura and a decrease in dural elastic fibers. Biondi et al. also agreed that recurrent subclinical neck trauma increases the risk of cord injury in patients by causing microcirculatory disruptions during physical activity [11].

Though family history in patients with HD has been insignificant, a few susceptibility genes, including C5orf42 and KIAA1377, also known as centrosomal protein 126 (CEP 126), were discovered by whole exome sequencing in four Korean patients [12]. HD is unlikely to occur in those who carry a single copy of the causative variation in either gene. A substantial correlation was found between the onset of the disease and either homozygosity for the CEP 126 mutation or heterozygosity for both variants. The genes CEP 126 and C5orf42 may act harmoniously as susceptibility genes for the development of monomeric amyotrophy [12]. Disease susceptibility may also be influenced by certain environmental factors, ethnic origin, or cultural and behavioral practices.

The Huashan criteria for diagnosis of HD [13] have been described in Table 1.

**Table 1:** Diagnostic criteria for Hirayama disease Definite Hirayama disease should meet clinical criteria 1, 2, and 3; imaging criteria 1 or 2; and NCS criteria 1, 2, and 3. Adapted from Yoshiyama et al. [10]

Clinical criteria	Imaging criteria	NCS criteria
1. Pubertal onset (in males)	1. Thinning of lower and middle cervical cord on neutral and flexion MRI	1. Neurogenic changes in AHC and/or radicles of middle and lower cervical cord
2. Localized wasting and weakness of upper limbs especially in the ulnar aspect of forearms and intrinsic hand muscles unilaterally or predominantly on one side	2. A crescent-shaped mass or loss of dural connection at the posterior epidural region on T2 weighted MRI	2. Conduction velocity in upper limb nerves: normal or mildly abnormal
3. No involvement of cranial nerves and lower limb muscles	3. Forward displacement and flattening of the lower cervical cord, narrowing of the anterior spinal cord on flexion MRI	3. No obvious cranial nerve and thoracic, lumbar, or sacral spinal cord involvement
4. Cold-induced paresis and tremors in fingers	4. High-intensity signals in anterior horns on T2 weighted imaging	
5. Mild sensory abnormalities in upper limbs	5. Straight alignment of cervical spine on X-ray	
6. Active deep tendon reflexes		

Investigations

Most cases of HD occur in adolescents, and early diagnosis is crucial for preventing long-term complications. X-rays of the cervical spine, myelography, neutral and flexion position MRI, electrodiagnostic studies, and motor unit number estimation (MUNE) are commonly done investigations for patients with suspicion of HD.

In flexion position, an MRI of a patient with HD usually shows atrophy, cord flattening, forward displacement of the cervical dural sac's posterior wall, and a crescent-shaped, high-intensity mass. Tightness of the dural sac during neck flexion is caused by the disproportionate distance between the vertebrae and their contents due to the juvenile growth spurt, the suspended dura mater anchored only at C2-C3, and the coccyx. This causes the cervical dural sac's posterior wall to move forward, followed by the cervical spinal cord moving forward and flattening. Consequently, ischemia and necrosis of AHC are brought on by microcirculatory disturbances in the anterior spinal artery-serving region forced on by prolonged compression; as a result, the cord appears thin owing to atrophy. MRI in a neutral position revealed focal areas of cord atrophy in the lower cervical and cervicothoracic junction as asymmetric flattening of the cord with the areas of gliosis appearing hyperintense on T2-weighted images.

Electrophysiological studies are essential in the diagnosis of HD. Segmental neurogenic injury of the AHC in the lower cervical spinal cord, without abnormalities of the sensory neurons, is the most significant finding on electromyoneurography (EMNG). Neurogenic lesions denoted by fibrillations and positive sharp waves are seen in patients with short duration of active disease. Large potentials caused by denervation and re-innervation and decreased MUNE are seen in patients with a longer disease duration. The progression of the disease is indicated by the occurrence of spontaneous potentials. Additionally, in patients with unilateral symptoms, subclinical disease may be picked up by electrophysiological studies on unaffected limbs. Motor nerve conduction reveals delayed latencies and reduced amplitudes in the afflicted upper limb muscles without aberrant conduction velocities. Sensory nerve conduction studies are usually normal. F-waves in patients affected with HD showed decreased frequency and conduction velocity but a normal latency period. A study by Zheng et al. [14] reported an increase in the latency period, whereas a study by Hassan et al. [5] found F-wave conduction velocities to be normal. Zheng et al. [14] also reported a significant increase in the percentage of median repeater F-waves. The phenomenon can be attributed to the reinnervation of the distal nerves through the sprouting of axons. Additionally, the application of stimulation that exceeds the maximum threshold selectively activates the larger motor neurons responsible for generating the F waves during neck flexion.

Functional imaging is an important consideration for future research. The Brownian motion of water particles is the basis for diffusion tensor imaging (DTI), a technique used to diagnose HD [15]. Possible markers are an increased apparent diffusion coefficient and a decreased fractional anisotropy. DTI is a useful tool for assessing injuries and can assist in guiding the degree of cord injury. The study found ipsilateral motor cortex activation when comparing the affected hand to the unaffected hand. The activation was reduced after surgical repair.

Patients suffering from HD have been investigated for somatosensory (SEP) and motor-evoked potentials (MEP) [16]. MEP has been linked to a reduced amplitude, an extended central motor conduction time, and a delayed latency. The upper limb amplitude of MEPs on cervical stimulation was significantly lower in multiple investigations, albeit inconsistently, when compared to the neutral position. In contrast, no such difference was noted between cervical stimulation and neutral position with SEP in patients with HD [17].

Treatment

Measures aimed at halting the progression of the disease constitute the most important part of treatment. Application of a cervical neck collar in the early stages of the disease for three to five years provided a good response. Monitoring the latency and amplitude of motor-evoked potentials guides deciding when to stop the cervical collar therapy. A favorable prognosis is observed in patients with a short duration of illness and mild or no cervical cord atrophy. Surgical fixation is aimed at relieving the compression of the cervical spine though it is used only in severe cases that are progressing quickly. The current guidelines suggest a surgical intervention when the disease continues to progress even after a long period of cervical collar therapy or in patients who cannot tolerate wearing a cervical collar.

Masaki et al. [18] believed increasing malalignment of the cervical spine was the primary cause of the disease. They performed a posterior C2-C6 cervical fusion on a patient after which the patient showed signs of regaining muscle strength. Similarly, Kohno et al. [19] performed C4-C5 cervical decompression and fusion on three patients with the disease. The disease progression was halted by successful surgery. Goel et al. [20] hypothesized that cervical spine alignment imbalance in HD patients extends beyond the afflicted segment to numerous levels and operated on four patients using the C1-C6/C7 posterior fixation. After surgery, the patient's post-operative symptoms did not progress.

Anterior surgical approaches in HD have been discussed in Table 2.

**Table 2:** Anterior surgical approaches in Hirayama disease Adapted from Gomathy et al. [2].

Surgical Approach	Description
Anterior cervical corpectomy and discectomy with plating	Surgical removal of vertebral body or disc from the front of the neck with stabilization using a plate
Anterior cervical plating only	Placement of a plate to stabilize the cervical spine without disc or vertebral body removal
Anterior cervical discectomy and fusion without plating	Removal of disc material followed by fusion of adjacent vertebrae without using a plate
Anterior cervical discectomy and fusion without plating	Removal of disc material followed by fusion of adjacent vertebrae without using a plate

Posterior surgical approaches in HD have been discussed in Table 3.

**Table 3:** Posterior surgical approaches in Hirayama disease Adapted from Gomathy et al. [2]

Surgical Approach	Description
Laminectomy, coagulation of epidural venous plexus with or without duraplasty	Removal of lamina (part of vertebra) and coagulation of veins in the spinal canal with or without a patch to enlarge the dural sac
Laminectomy and fixation	Removal of lamina with stabilization using screws and rods
Posterior uninstrumented fusion	Fusion of vertebrae in the cervical spine without additional hardware
Posterior cervical laminoplasty and duraplasty	Expansion of the spinal canal by creating a hinge on one side of the lamina and patching the dura mater
Posterior fixation only	Stabilization of the cervical spine using screws and rods without removing lamina

## Conclusions

HD is a motor-predominant neuropathy affecting the upper limbs in young Asian adolescents. The disease is most commonly seen in growing adolescents due to the spinal cord outgrowing the available space in the vertebral column. Although it commonly presents as asymmetrical involvement of a single upper limb, bilateral upper limb involvement is also possible. Diagnosis is usually made using MRI in flexion and neutral positions, along with nerve conduction studies. Treatment aims to reduce traction on the spinal cord by avoiding spinal flexion. Wearing a cervical neck collar typically halts disease progression. Surgical interventions are usually considered for those who do not achieve sufficient symptom control with a cervical collar. Further studies on genetic and environmental associations are needed to better understand the disease pathology and to develop more effective treatments.
